# Comparative analysis of physiological variations and genetic architecture for cold stress response in soybean germplasm

**DOI:** 10.3389/fpls.2022.1095335

**Published:** 2023-01-06

**Authors:** Muhammad Azhar Hussain, Senquan Li, Hongtao Gao, Chen Feng, Pengyu Sun, Xiangpeng Sui, Yan Jing, Keheng Xu, Yonggang Zhou, Wenping Zhang, Haiyan Li

**Affiliations:** ^1^ Sanya Nanfan Research Institute of Hainan University, Hainan Yazhou Bay Seed Laboratory, Sanya, China; ^2^ College of Tropical Crops, Hainan University, Haikou, China; ^3^ College of Life Sciences, Jilin Agricultural University, Changchun, China

**Keywords:** cold stress, RNA-seq, miRNA, photosynthesis, antioxidants, soybean

## Abstract

Soybean (*Glycine max* L.) is susceptible to low temperatures. Increasing lines of evidence indicate that abiotic stress-responsive genes are involved in plant low-temperature stress response. However, the involvement of photosynthesis, antioxidants and metabolites genes in low temperature response is largely unexplored in Soybean. In the current study, a genetic panel of diverse soybean varieties was analyzed for photosynthesis, chlorophyll fluorescence and leaf injury parameters under cold stress and control conditions. This helps us to identify cold tolerant (V100) and cold sensitive (V45) varieties. The V100 variety outperformed for antioxidant enzymes activities and relative expression of photosynthesis (*Glyma.08G204800.1*, *Glyma.12G232000.1*), GmSOD (*GmSOD01*, *GmSOD08*), GmPOD (*GmPOD29*, *GmPOD47*), trehalose (*GmTPS01*, *GmTPS13*) and cold marker genes (*DREB1E*, *DREB1D*, *SCOF1*) than V45 under cold stress. Upon cold stress, the V100 variety showed reduced accumulation of H_2_O_2_ and MDA levels and subsequently showed lower leaf injury compared to V45. Together, our results uncovered new avenues for identifying cold tolerant soybean varieties from a large panel. Additionally, we identified the role of antioxidants, osmo-protectants and their posttranscriptional regulators miRNAs such as miR319, miR394, miR397, and miR398 in Soybean cold stress tolerance.

## Introduction

Soybean (*Glycine max* L.), one of the largest sources of edible plant oil and protein, have the capability to feed the ever-increasing world population. After originating in East Asia (China), soybean cultivation begins worldwide (Zhou et al., 2015; Schulte et al., 2017). Currently, China is one of the largest producers, consumers and importers of soybean. Soybean is cultivated at different latitudes and altitudes in China. Being tropical in nature, soybean is sensitive to cold (Cheesbrough, 1990), which hampered the soybean production area in China. Depending on the cultivation area, cold stress affects the germination, seedling and reproduction stages of soybean (Ohnishi et al., 2010). Admittedly, a healthy seedling stand is one of the most critical stages in the plant life cycle; it determines the good final yield and quality of cultivated crops. Due to unexpected weather conditions, cold stress at the seedling stage caused irreplaceable production loss of soybean in prominent growing areas (Cheng et al., 2010). Generally, in plants, cold stress results in low germination rates, growth retardation, tissue injures, physiological changes, such as antioxidant metabolism activation and metabolic perturbation, and reduced photosynthetic efficiency (Mehrotra et al., 2020; Gao et al., 2022).

In green plants, the photosynthesis process utilizes light energy and converts it to carbohydrates. Chlorophyll (Chl) contents (SPAD index) and fluorescence is an important indicators of photosystem II (PSII) efficiency. PSII is described as the ratio of Fv to maximal fluorescence emission (Fv/Fm) (Logan et al., 2007; Adams et al., 2013). Cold stress severely damaged the chloroplast structure and chlorophyll content in susceptible sugarcane cultivars which was reduced to lower levels than that in cold-resistant cultivars (Zhang et al., 2015; Li et al., 2018). In response to environmental stresses, plants protect the photosynthetic apparatus through photoinhibition, managing the Fv/Fm, dissipation of light energy as heat and accumulation of osmolytes such as sugars (Adams et al., 2013). Cold stress inhibits stomatal opening and causes a reduction in stomatal size which further impairs the photosynthesis efficiency. In young soybean seedlings, cold stress actively reduces the photosynthesis capability and the effect is more severe during higher illumination (Balestrasse et al., 2010). The Fv/Fm values has been used as nondestructive methods to measure photosynthetic activity under cold stress (He et al., 2019; Mehmood et al., 2021). Cold tolerant varieties maintained higher photosynthesis efficiency by protecting the photosynthesis apparatus which is widely used indicator for cold tolerant germplasm screening which needs to be determined in soybean. During photosynthesis, light energy is captured by light-harvesting complexes (LHCs) located in the thylakoid membrane of chloroplasts and used to drive photochemistry. However, excessive or a fraction of absorbed light through LHCs is lost through non-photochemical quenching (NPQ) as heat or emitted as chlorophyll fluorescence (Bassi & Dall'Osto, 2021; De Souza et al., 2022). The NPQ plays several important roles in protecting the cell organ during photosynthetic processes by dissipating excessive energy and preventing the accumulation of reactive intermediates (Müller et al., 2001; Tietz et al., 2017). For instance, tomato plants treated with Melatonin showed rapid increase in NPQ value to ameliorating the cold effects and to protect the photoinhibition process (Ding et al., 2017). Therefore, differences in NPQ values could be used to identify the soybean varieties with higher cold tolerant capabilities.

Arabidopsis species having the potential to increase the leaves’ thickness under cold stress conditions exhibited higher photosynthesis capacities due to the presence of more significant number of phloem cells and efficient loading and unloading of sugars (Stewart et al., 2016). Therefore, leaf thickness is a nondestructive trait and could be used as a potential trait to identify the cold tolerant soybeans with higher photosynthesis capability. The optimum temperature for leaf-scale maximum photosynthesis varies within plant species, leaf age, growing climates and soil nutrient conditions (Liu, 2020). The immediate effects of temperature fluctuation in photosynthesis efficiency at the leaf-scale were widely studied. Under cold conditions, leaf-scale photosynthetic carbon dioxide assimilation is reduced by the maximum carboxylation rate of Rubisco (Rogers et al., 2017). Plant varieties having the capability to maintain the leaf temperature under normal ranged under stress condition seems to be more stress resilient especially under cold stress conditions.

At the genetic level soybean plant respond to cold stress by activating the various cold-responsive genes including *GmDREB3* (Chen et al., 2009), novel MYB transcription factor *GmMYBJ1* (Su et al., 2014), *GmDREB1* (Yamasaki & Randall, 2016), NIMA-related kinase *GmNEK1* (Pan et al., 2017) and mitochondrial calcium uniporter GsMCU (Li et al., 2022). The *GmSCOF1* genes responded to cold stress conditions and have been functionally validated by stable ectopic overexpression in Arabidopsis (Liao et al., 2008). However, due to unresponsiveness to the cold acclimation process, the soybean plant showed cold sensitivity. The main possible genetic reason for soybean cold sensitivity could be explained as cold acclimation-related genes are grouped and located on the same chromosome in Arabidopsis and tomato plants, but these genes are scattered in the soybean genome and located on different chromosomes (Yamasaki & Randall, 2016). Therefore, it is worthy to explore new cold stress-responsive pathways in addition to COR pathways in soybean. Accumulation of different antioxidants, metabolites and osmo-protectants to ameliorate the cold stress effects are additional approaches adopted by plants. Antioxidant enzymes SOD, POD and osmo-protectants like trehalose accumulation have been proved to their soothing effects in response to cold stress in Arabidopsis, rapeseed, rice, watermelon and maize (Kosar et al., 2019; Liu et al., 2021; Raza et al., 2022). However, their understandings remained unexplored especially under cold stress conditions in soybean. Being genetically controlled, the posttranscriptional regulations of SOD, POD and trehalose pathways remained to be disclosed in soybean. The miRNAs have been proven as the posttranscriptional regulator of genes and play critical roles in stress response pathways (Cui et al., 2017). The miRNA-mediated genetic pathways such as miR397-*LACCASE*, miR394-*LCR* and miR319-*PCF/TCP* have been functionally validated for their significant cold stress response in plants (Dong and Pei, 2014; Wang et al., 2014; Song et al., 2016). The identification of antioxidant and metabolites genes and their posttranscriptional regulators will help to identify novel cold stress pathways in soybean.

Soybean breeders are striving for the identification of genetic resources and efficient breeding strategies for the development of biotic and abiotic stress-responsive (cold), improved quality and higher yielding soybean varieties (Ray et al., 2013). Therefore, a foremost task to speed up the soybean breeding program is a global dissection of the physiological and genetic basis of important traits (Fang et al., 2017). We hypothesize that the physiology of the natural population of soybean responds differently to cold stress. Thus, the understanding of the physiological mechanisms developed by soybean to thrive under cold conditions will be an enormous source of information for soybean breeders in identifying potential cold-tolerant genotypes. Therefore, one of the objectives of this study was to evaluate the physiological performance of a large soybean population grown under normal and cold stress conditions. Secondly, this study identified the cold tolerant and cold sensitive genotypes and their molecular and genetic differences for cold stress. Our results provide new insights of the nondestructive physiological mechanism that responds to cold condition in soybean. In addition, the traits selected in this study will be helpful for marker-assisted breeding and genome wide association studies to identify candidate QTLs and genes to develop cold stress resilient soybean.

## Material and methods

### Plant material and growth conditions

The experimental germplasm was obtained from Soybean Research Group, Sanya Nanfan Research Institute (SNRI), Hainan University, China. The experiment was replicated twice, in September–October, 2021 (College of Tropical Agriculture, Hainan University, Haikou) and January–February, 2022 at SNRI, China, under controlled growth chambers (phytotrons) individually at both locations. The research panel contained 100 diverse soybeans (*Glycine max* L.) (**Table S1**). These Soybean cultivars were grouped based on their cultivation area (higher or lower latitude), domestication area and final consumption as vegetable, protein source and edible oil into vegetable group (V group), SD group, KC group and C group. These varieties are highly diverse in terms of plant architecture, plant height, leaf size, leaf color, branch angle, pod length, number of seeds per pod, seed size and weight, seed color and luster, earliness, yield and related traits. The soybean seeds were stored at a working temperature of 10°C in yellow paper bags to protect them from light and moisture; for seed sowing, peat soil was prepared by mixing three parts of vermiculate and one part of compost soil and filled in 3 × 3 inches black plastic pot. The pots were arranged following the complete randomization design (CRD) in 3 replications and irrigated with tap water to moist the soil. Selected seeds were free from chemical treatments such as antifungal dressing. Three seeds per pot were sown at a uniform depth in each pot and covered with a plastic tray cover. Plastic trays were transferred to a growth chamber with the following controlled environmental conditions; photoperiod 12/12 h day and night, constant 22°C temperature, 60% relative humidity and 20000 lux light. After seed germination, each pot tray was fertigated every third day. Seedlings were allowed to grow under these growth conditions until they reached the V2 leave stage (2^nd^ trifoliate).

### Cold stress treatment

For cold stress treatment, the growth chamber (phytotron) day/night temperature was reduced to 4°C, and healthy and uniform soybean seedlings were selected and transferred to cold stress conditions for five days. In phytotrons, relative humidity 60%, photoperiod 12/12 h day/night and light flux intensity 20000 lux was maintained as for normal growth conditions.

### Data collection

Under control growth conditions data were collected from six individual plants. As V1 stage leaves were fully developed compared with V2 stage leaves therefore selected for data collection. Under cold stressed conditions data were collected from five to six plants from each replication and average data of each replication was further used. Leaf area were recorded and analyzed as described previously (Fang et al., 2017) and leaf thickness was recorded with the vernier calipers.

### Estimation of PSII photosynthetic efficiency studies

The PSII photosynthetic efficiency under control and cold stress conditioned soybean leaves was determined from the chlorophyll fluorescence analysis of photochemical yield (Fv/Fm), using a portable chlorophyll-fluorometer OS-30p^+^ (OPTI-SCIENCES, China).

### Chlorophyll fluorescence

The qL, PhiNO, and NPQt traits data under control and cold stressed soybean leaves were recorded using MultispeQ 2.0 (PHOTOSYNQ, RoHS, USA). MultispeQ 2.0 device was remotely connected with mobile phones application through Bluetooth and data was immediately stored in an online database (https://photosynq.org). Data was retrieved by accessing the above-given website and analyzed to evaluate the performance of under-study germplasm.

### Identification of GmPOD, GmSOD and GmTPS genes and their sequence data retrieval

The soybean genome assembly *G. max* Wm82.a2.v1 was accessed through Phytozome 13 database (https://phytozome-next.jgi.doe.gov/) to retrieve the coding sequences of all GmPOD, GmSOD and GmTPS genes by blasting the Arabidopsis sequence for these genes as the query sequence. Subsequently, all predicted sequences were further validated by BLAST search using online available conserved domain (CDD) databases and protein family (Pfam) databases (Marchler-Bauer et al., 2015; El-Gebali et al., 2019).

### Expression patterns of GmPOD, GmSOD and GmTPS family genes based on transcriptome sequencing data

To analyze the expression profiles of GmPOD, GmSOD, GmTPS and photosynthesis related genes, the soybean transcriptome expression data was used from the NCBI database with accession number GSE117686 submitted under control, 1h and 24h cold stress conditions (Yamasaki and Randall, 2016). The gene expression value for each gene was obtained from an online resource PPRD (http://ipf.sustech.edu.cn/pub/plantrna/) (Yu et al., 2022).

### Quantification of POD and SOD enzymes activities, H_2_O_2_ content, and MDA level

The POD and SOD, H_2_O_2_, and MDA activity levels were measured under control, 1h and 24h cold stress in soybean leave samples stored at -80°C. The activities of POD and SOD, H_2_O_2_ content, and MDA accumulations were examined using a peroxidase kit id: BC0090 (SolarBio, Beijing, China), superoxide dismutase kit id: BC0175 (SolarBio, Beijing, China), H_2_O_2_ test kit id: BC3595 (SolarBio, Beijing, China), and MDA test kit id: BC0025 (SolarBio, Beijing, China) according to the manufacturer protocols. All measurements were performed with three independent replicates.

### Prediction of the 3D protein structure of photosynthesis and GmSOD proteins

The predicted 3D protein structures of photosynthesis and GmSOD genes, amino acid sequence was blasted in the EBI Protein Similarity Search tool (https://www.ebi.ac.uk/Tools/sss/fasta/) and respective DB : ID was searched in AlphaFold Protein Structure Database (https://alphafold.com/search/text/). The PDB file was retrieved from AlphaFold Protein Structure Database and ChimeraX 1.4 (https://www.cgl.ucsf.edu/chimerax/) was used to visualized protein structure.

### Prediction of putative miRNA targeting GmPOD, GmSOD and GmTPS genes and GO annotation analysis

The coding sequence (CDS) of GmPOD, GmSOD and GmTPS family genes were blasted in the psRNATarget database (http://plantgrn.noble.org/psRNATarget/) by default mode to predict the posttranscriptional regulation of genes by miRNAs (Dai et al., 2018). The miRNA and predicted targeted genes interactive network were visualized by Cytoscape software (V3.8.2; Available online: https://cytoscape.org/download.html). Gene ontology (GO) annotation analysis was performed by uploading all GmPOD, GmSOD and GmTPS protein sequences to the eggNOG website (Available online: http://eggnog-mapper.embl.de/, 1 July 202) (Powell et al., 2014). An R-package GO-plot (https://wencke.github.io/#circular-visualization-of-the-results-of-gene-annotation-enrichment-analysis-gocircle) was used to perform GO enrichment analysis and data visualization.

### Selection index for cold tolerance

The optimum selection index (Falconer, 1967) was calculated for cold tolerance based on high heritability and phenotypic and genotypic correlation. Cold tolerance index (CTI) was measured using the correlated response between leaf injury index (X1), qL (X2) and Fv/Fm (X3) traits:

CTI = b1X1 + b2X2 + b3X3

Where, b1 = 0.967, b2 = 0.505, b3 = 0.335

Here, b1, b2, and b3 represent the index coefficients. The vector of SmithHazel index coefficient b was measured as previously reported (Baker, 1986).

b = P ^− 1^ G,

where P^-1^ represents the inverse of the observed phenotypic variance-covariance matrix for the selected traits; G is a matrix involving the measurements of genotypic and covariance. The low CTI values indicated higher cold tolerance.

### RNA extraction and qRT-PCR analysis

Total RNA was extracted from tissue samples stored at -80°C using a plant total RNA extraction kit (Takara, Japan) following the manufacturer’s instructions. Total RNA quality and quantity were measured using a NanoDrop 2000 spectrophotometer (Thermo Fisher Scientific, West Palm Beach, FL). Approximately lg total RNA was used for cDNA development using one step cDNA synthesis kit (Tranz, China) according to the manufacturer’s instructions. The qRT-PCR was conducted as previously described for selected genes (He et al., 2019). Genes primers were designed using NCBI-Primer blast webserver, and primers are listed in **Table S2**. Endogenous Actin gene *Glyma.02g091900* (Liu et al., 2016) was used for relative control and three technical or biological replicates were performed per experiment.

## Results

### Principal component analysis of phenological and physiological traits

**Figure 1:**
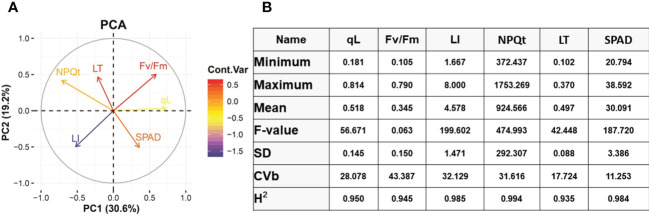
PCA analysis and Basic statistical analysis. **(A)** PCA analysis of different traits. **(B)** Analysis of Variance (ANOVA). qL, Fv/Fm, Non-photochemical quenching (NPQt), LI= leaf injury, LT=Leaf thickness, SD= standard deviation, CVb= coefficient of variation, H^2^= Heritability, GA= Genetic advance.

A panel of 100 soybean varieties grown in 2021 and 2022 were used in this study (**Table S1**). Different traits related to photosynthesis and leaf injury index were investigated under 22°C control and 4°C cold stress (**Figure 1A**). PCA analysis was performed to explore the relationships among six trait variables and the source of trait variation. According to the results, PC1 explained 30.6% and PC2 explained 19.2% of the trait variance individually and presented 49.8% of the accumulative variance in the dataset (**Figure 1A**). Interestingly, traits negatively affected by cold stress are clustered nearby such as qL and Fv/Fm and, on the contrary, NPQt and leaf thickness increased their values under cold stress are clustered in the same coordinate (**Figure 1A**). The separation of these traits in different coordinates in PCA indicated that cold stress significantly impacted the studied traits in soybean. The qL and FV/Fm variables of PCA are positively correlated, and both have a significant negative correlation with leaf injury index and NPQt which were aligned on PC2 (**Figure 1A**). The SPAD traits loaded on PC1 remained independent of other traits under cold stress. The PCA results suggested that PC1 and PC2 can be used as quantitative indices to characterize or identify cold tolerance varieties, respectively.

### Genetic variation for cold tolerance at the seedling stage in soybean

**Figure 2:**
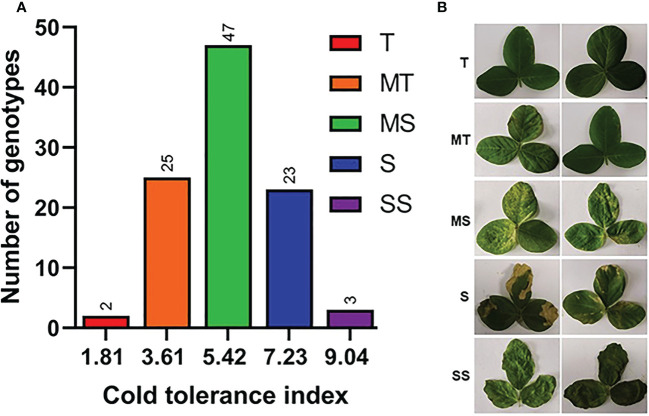
Cold tolerance selection index in diverse Soybean germplasm. **(A)** Cold tolerance indexes, **(B)** Phenotypic categorization, T, tolerant; MT, moderate tolerant; MS, Moderate sensitive; S, sensitive; SS, severely sensitive. Phenotype was analyzed after three days of recovery.

To explore the genetic variation among Soybean genotypes, we performed analysis of variance (ANOVA) based on physiological and phenotypic data (**Figure 1B**). The ANOVA results revealed that soybean varieties significantly differ (P < 0.01) for all cold stress-related traits. Heritability analysis (H^2^) revealed that these traits had high heritability >90% most probably due to similar behavior of traits under cold stress conditions (**Figure 1B**). This higher heritability behavior of traits also indicates that the difference is entirely due to genotypic variations and the minute role of environmentally-caused variations. The phenotypic variation between varieties in cold tolerance index (CTI) is shown in (**Figures 2A** **, B**). The CTI analysis distributed the varieties into five distinct categories based on their cold tolerance capability: tolerant (2 varieties), moderate tolerant (25 varieties), moderately susceptible (47 varieties), susceptible (23 varieties) and severely susceptible (3 varieties). The vegetable soybean variety V100 (CTI = 1.81) was found cold tolerant variety compared to vegetable soybean variety V45 which was the most susceptible (CTI > 8). These results indicate that the symptoms of leaf injury increased gradually with the increment of cold duration and resulting decrease in qL and Fv/Fm index in all soybean varieties.

### Evaluation of chlorophyll fluorescence qL parameter in soybean

**Figure 3:**
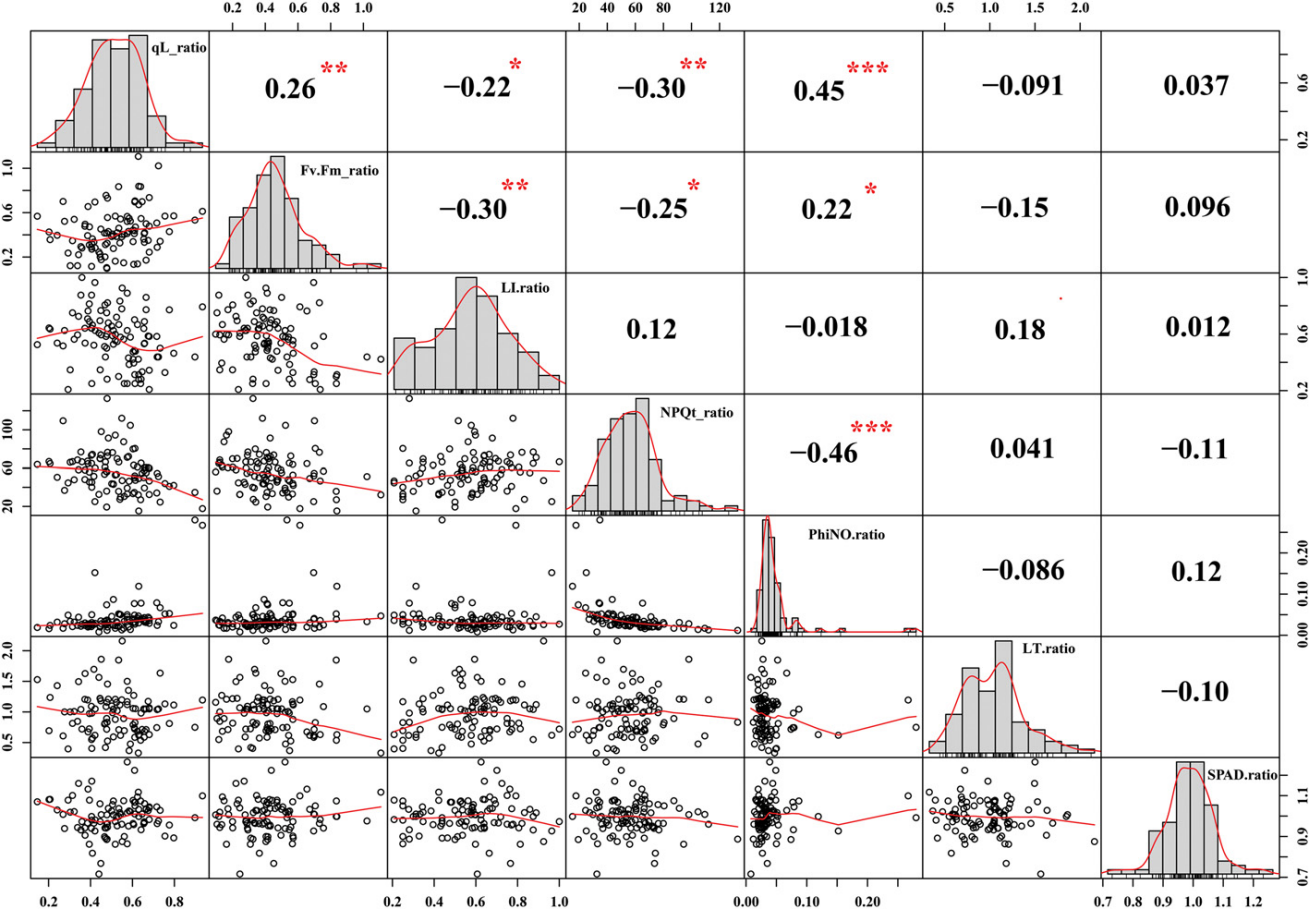
Correlation analysis of different evaluated traits in Soybean. Traits for correlation analysis: qL ratio = qL_Cold/qL_CK, Fv/Fm ratio = Fv/Fm_Cold/Fv/Fm _CK, Leaf injury ration (LI) ratio = LI_Cold/LI _CK, Non-photochemical quenching (NPQt) ratio = NPQt_Cold/NPQt_CK, phiNO ratio = phiNO_Cold/phiNO _CK, Leaf thickness (LT) ratio= LT_Cold/LT_CK, and SPAD ratio= SPAD _Cold/SPAD _CK. The ***, ** and * asterisks indicate the significant correlation at p-value <0.001, <0.01 and p-value <0.05, respectively.

Chlorophyll fluorescence is a non-destructive quantification of photosystem II (PSII) activity and is widely used to understand the photosynthetic mechanisms in response to abiotic and biotic stresses (Murchie and Lawson, 2013). The qL parameter is known as the coefficient of photochemical quenching. In previous research, differences in the qL parameter were exploited to understand the photosynthesis response mechanism under cold stress (Acidri et al., 2020). In our data, we have observed that cold stress reduced the qL value compared to control conditions in Soybean germplasm. However, the reduction was more abrupt in cold-sensitive Soybean varieties compared to tolerant. We reported qL values under control conditions (0.963 to 0.993) and cold stress (0.181 to 0.814) conditions (**Figure 1A**). As qL value was measured through a non-destructive method from plant leaves, therefore, we correlated the qL values with leaf injury levels. As shown in **Figure 3**, the qL value has a significant negative correlation (r^2^= -0.22) with leaves injury level under cold stress conditions. These results supported our hypothesis that the differences in qL value are a useful indicator of cold tolerance evaluation of a large population to identify the stress-resilient germplasm of soybean.

### Evaluation of chlorophyll fluorescence parameter Fv/Fm in soybean

Cold stress signaling above freezing temperature triggered various physiological processes to ameliorate the deleterious effects on plant cellular organs, referred to as cold or chilling acclimation. Among the immediate physiological response, cold stress reduced the chlorophyll fluorescence trait (maximum quantum efficiency of PSII) (Fv/Fm) in the dark-adapted state and impaired the photosynthesis process (Gao et al., 2022). Similarly, we observed a significant reduction in Fv/Fm under cold stress conditions from 0.105 to 0.790 compared to control conditions from 0.719 to 0.883 (**Figure 1B**). On average acute reduction of Fv/Fm in our cold-sensitive germplasm indicates severe PSII photoinhibition under cold stress conditions compared to cold tolerant germplasm (**Figure 1A**). Similarly, in a previous study, in response to freezing stress, transgenic *Populus euphratica* overexpressing PeSTZ1 maintained higher photosynthetic activity (Fv/Fm) by dissipating more excessive light energy which imparts them higher freezing tolerance compared with wild type (He et al., 2019). These results indicate that the maximum quantum efficiency of PSII in the dark-adapted state (Fv/Fm) could be an essential parameter for screening the cold tolerant germplasm (Gao et al., 2022). Different studies reported that cold stress led to cold injury to plants’ organs, including leaves which were directly facing the environmental anomalies and exhibited the cold effects directly. Being Fv/Fm is a widely accepted index for plant stress tolerance evaluation, such as cold/freezing (Ehlert and Hincha, 2008; He et al., 2019), pathogen (Rousseau et al., 2013), drought (Woo et al., 2008), and heat (Xu et al., 2014). Therefore, to identify the association between Fv/Fm and leaves injury level parameters, we performed a correlation analysis. According to the results, Fv/Fm has a significant negative correlation (r^2^=-0.30) with leaves injury levels under cold stress (**Figure 3**). The higher correlation under cold stress indicates that the reduction of Fv/Fm caused an imbalance in the redox state and overproduction of reactive oxygen species which leads to cellular leaves injury and death. Thus, we selected Fv/Fm as one of the parameters for further analysis and screening of cold tolerant and cold sensitive germplasm of soybean.

### Evaluation of chlorophyll fluorescence NPQt parameter in soybean

Abiotic stresses acutely inhibit photosynthetic efficiency but increase the nonphotochemical quenching (NPQ) capability of stressed plants (Jahan et al., 2021). When plants faced extreme temperature stress, a surging in P700 oxidation immediately repressed the quantum efficiency of PSII (Y (II)), which directly caused an escalation of NPQ, the decline in the quantum efficiency of PSI and a reduction in the plastoquinone pool (Y(I)) (Wada et al., 2019). In order to determine the role of NPQt in the cold stressed soybean, the change in NPQt was measured compared to control conditions. We observed NPQt values under control conditions (7.652 to 42.576) and cold stress (372.437 to 1753.269) conditions (**Figure 1B**). This data indicates that under cold stress, NPQt changed vastly, and considerable variability among germplasm for NPQt value showed that the response of each soybean variety is different under cold stress conditions. According to our correlation analysis, NPQt has a significant negative correlation with qL and Fv/Fm parameters and has a positive but insignificant association with leaf injury index (**Figure 3**). These results indicate that the reduction of qL and Fv/Fm reduced the photosynthesis capability in cold-sensitive varieties and plants responded by increasing the NPQt to dissipate the excessive light energy to protect the leaf damage in soybean. Thus, NPQt levels could be used to differentiate the cold-sensitive and cold-tolerant germplasm of soybean.

### Evaluation of leaf injury index and leaf thickness in soybean

Leaves are the major photosynthetic organ responsible for energy harvesting and conversion into life energy and directly face environmental challenges: flavonoids and sugar abundance in leaves and stems are linked with cold stress tolerance. Flavonoids are the product of the phenylpropanoid metabolic pathway (Sharma et al., 2007). In previous studies, chilling stress tolerant maize showed thicker cell walls, mesophyll layers and bundle sheath cells. Additionally, tolerant plants showed less effected Fv/Fm and photosynthetic-related traits compared with the cold-sensitive plant (Bilska-Kos et al., 2018). Thus, leaves are plants organs which are directly affected and exhibit the visible stress effects in the form of injuries in response to environmental anomalies including cold stress. Leaves injury index level and leaf thickness is widely used for the screening of cold tolerance germplasm (Bilska-Kos et al., 2018; Zeng et al., 2021). Therefore, we adopted leaves injury index and leaf thickness along with other traits for screening the soybean cold tolerant material. According to our results, leaf injury index ranged under cold stress from minimum 1.67 to maximum level 8 and leaf thickness 0.102 mm to 0.370 mm compared with 0.10 mm to 0.46 mm control conditions (**Figure 1B**). This data indicates that cold stress increased the leaf injury level in cold-sensitive varieties; however, cold tolerant varieties exhibited slightly higher leaf thickness along with the slightest leaf injury.

### Evaluation of SPAD values in soybean

Under stress, protection of Chlorophyll (Chl) content reflects the plant’s photosynthetic capacity and stress tolerance capability. The Chl content was quantified as SPAD value and depended on stress levels, genotypes and their growing environment (El-Hendawy et al., 2022). The abiotic stresses such as cold stress led to the overproduction of reactive oxygen species (ROS) which reduced Chl synthesis and accelerated Chl degradation in sensitive cultivars (Wang et al., 2016a; Acosta-Motos et al., 2017). Thus, measurement of leaf Chl content is an effective screening criterion for genotype screening to strengthen the breeding programs. Following previous studies, we estimated the SPAD value in the cold-stressed soybean cultivars and found large variability ranging from 20.794 minimum to 38.592 maximum (**Figure 1B**). However, we could not find a significant correlation of SPAD value with leaf injury index and other studied traits (**Figure 3**). These results indicate that SPAD value in soybean could be used as an individual selection criterion for screening instead of combined with other traits as previously used for screening freezing tolerance wheat cultivars (Wang et al., 2016a).

### Major differences for phenological and physiological traits in cold tolerant and sensitive varieties

**Figure 4:**
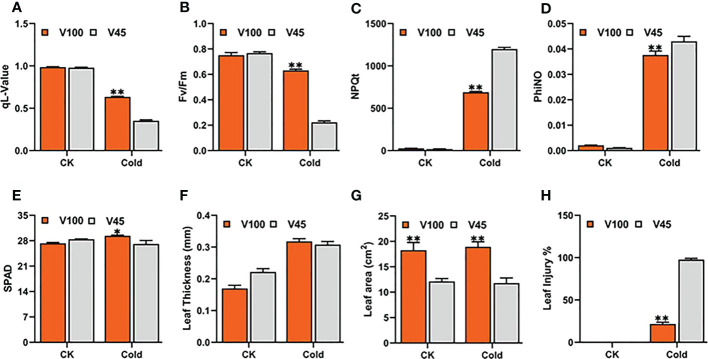
Physiological and phenological differences between V100 and V45. Traits were recorded under control 22°C and cold stress 4°C conditions. **(A)** qL-value, **(B)** Fv/Fm, **(C)** NPQt, **(D)** phiNO, **(E)** SPAD, **(F)** Leaf thickness (LT), **(G)** Leaf area, **(H)** Leaf injury (LI). Data was analyzed with the Statistix 8.1. The ** and * asterisks indicate the significant differences at p-value <0.01 and p-value <0.05*, respectively.

In order to choose the most promising soybean varieties with high cold tolerance at the seedling stage, the soybean varieties were categorized based on the direction of cold tolerance indexes (CTI) from most cold tolerant to susceptible in qL, Fv/Fm, NPQt and leaf injury levels (**Figure 2A**). Then, the two cold tolerant varieties (V100 and V037) and two cold-sensitive varieties (V45 and V004) significantly differed in four traits qL, Fv/Fm, NPQt and leaf injury levels were selected. Among these four, the cold tolerant V100 and cold sensitive V45 were selected for further analysis. We observed that under cold stress, V100 showed a higher qL value (0.63) compared to V45 (0.353) (**Figure 4A**). Similarly, V100 performed significantly better for Fv/Fm (0.631) in respective to V45 (0.219) (**Figure 4B**). The NPQt level was found lower in V100 (688.50) compared to cold-sensitive V45 (1199.23), which indicates that V100 maintained the lowest nonphotochemical quenching capability by maintaining the redox level and protecting the photosynthetic system from generated reactive oxygen species (**Figure 4C**). The levels of PhiNO were significantly lower in V100 which is also attributed to photosynthetic system protection (**Figure 4D**). The SPAD value and leaf thickness are slightly higher in V100 than in V45 but were not significantly correlated with the leaf injury index (**Figures 3**, **4E, F**). However, these traits could be used as individual traits as cold tolerance indexes but could not best fit in our Soybean genetic material. Hence, we did not include in our selection criteria to choose cold tolerance and cold-sensitive soybean. The leaf area is an important trait and is directly responsible for light energy capturing and plant food preparation. We compared the leaf area between V100 and V45. Our data indicate that the leaf area in V100 is 18.1 cm^2^ in contrast to V45 which is 12.3 cm^2^ (**Figure 4G**). However, no significant differences exist for leaf area measured under control and after cold stress conditions. We further compared the V100 with V45 based on leaf injury protection and found the lowest leaf injury 18% in cold tolerant compared to 98% in cold-sensitive variety (**Figure 4H**). These results indicate that cold stress stops the growth and development of soybean plants under cold stress conditions. However, the cold tolerant varieties prepared themselves to ameliorate the deleterious effects of cold stress by maintaining and protecting the photosynthesis system possibly by producing various kinds of metabolites and activating the antioxidant system. These findings indeed help to explain why V100 is more cold tolerant than V45.

### The expression differences of photosynthesis-related genes in V100 and V45

**Figure 5:**
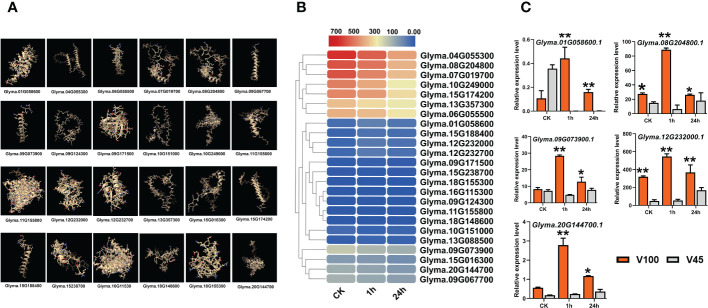
Protein structure and expression of photosynthesis related genes. **(A)** Predicted protein structure of photosynthesis genes, **(B)** RNA-seq expression analysis of photosynthesis genes under cold stress conditions, **(C)** qRT-PCR verification of photosynthesis genes in V100 and V45. Data was analyzed with the Statistix 8.1. The ** and * asterisks indicate the significant differences at p-value <0.01 and p-value <0.05, respectively.

To understand the expression behavior of photosynthesis-related genes in cold stress conditions in soybean. We performed a genome-wide survey and identified various essential photosynthesis-related genes. We predicted the 3D structure of photosynthesis-related genes in soybean (**Figure 5A**). The expression of these genes was explored using previous RNA-seq data under control, 1h and 24h of 4°C cold stress treatment in soybean (Yamasaki and Randall, 2016). As illustrated in the heatmap, indeed, cold stress treatment had reduced the expression of most of the photosynthesis-related genes in soybean (**Figure 5B**). These results verify our physiological data for qL, Fv/Fm and NPQt levels and indicate that these traits are genetically controlled under cold stress conditions in soybean. Therefore, we collected the tissue samples of cold tolerant V100 and cold sensitive V45 under control, 1h and 24h cold stress treatment to explore the variations at genetic levels. Our time course qRT-PCR analysis indicates that the expression of most of these photosynthetic genes was reduced at later stages in both varieties under cold stress (**Figure 5C**). However, the expression reduction of these genes was found more pronounced in V45 compared to V100. Additionally, some selected genes in V100 showed higher expression compared to previous RNA-seq data which might be due to differences in genetics or stress response mechanism of varieties used in experiments (**Figure 5C**). Altogether, in comparison with previous RNA-seq data and our time course, qRT-PCR results suggested that cold tolerant soybean varieties maintained their photosynthetic capability by regulating the expression of different photosynthesis-related genes. The expression differences of these genes could be used as a marker to identify the cold tolerance levels in soybean varieties.

### The role of antioxidant SOD, related genes and their post-transcriptional regulators in V100 and V45

**Figure 6:**
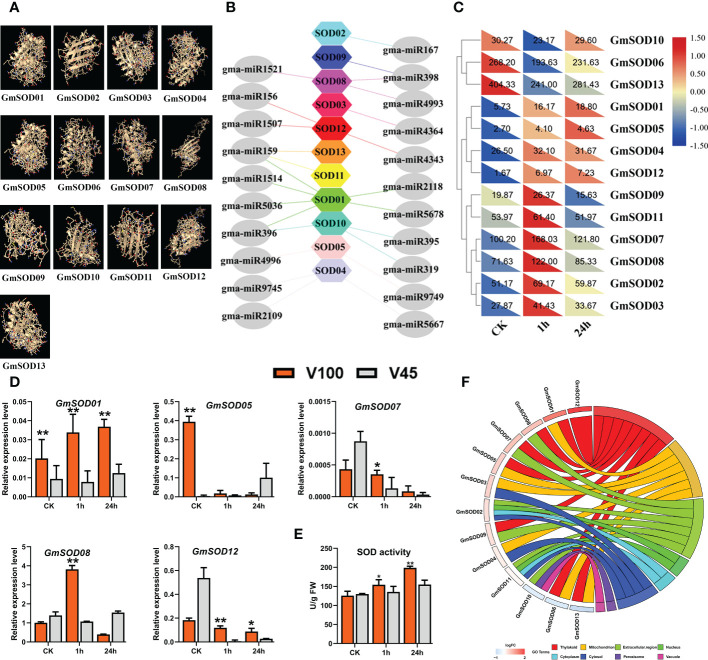
GmSOD genes and posttranscriptional regulation in V100 and V45. **(A)** Predicted protein structure of GmSOD genes in Soybean. **(B)** Post-transcriptional regulation of GmSOD genes by gma-miRNA. **(C)** RNA-seq expression analysis of GmSOD genes under **(CK)** and cold stress conditions. Expression data was normalized by log_2_FC. **(D)** qRT-PCR verification of GmSOD genes in V100 and V45. **(E)** SOD enzyme activity. **(F)** Gene ontology analysis of GmSODs. Data was analyzed with the Statistix 8.1. The ** and * asterisks indicate the significant differences at p-value <0.01 and p-value <0.05, respectively.

To encounter and eliminate the toxic ROS, plant cells have developed a series of antioxidant defense systems which equilibrate the oxidative reaction to protect the cellular damage (Geng et al., 2018). Among these enzymatic defense systems, superoxide dismutase (SOD) has a promising role in ROS scavenging under cold stress (Su et al., 2021). The SOD efficiently catalyzes the toxic O^2−^ free radicals, balances the ROS metabolism, and mitigates the stress effects to protect cell membrane structure (Das and Roychoudhury, 2014; Huang et al., 2022). To understand the role of SOD in cold tolerance in soybean we quantified SOD activity in V100 and V45 in control and cold stressed samples for 1h and 24h. Our SOD enzyme quantification assay revealed that SOD activity was increased under cold stress in both varieties compared with control conditions. However, we observed a quick increment of SOD enzymatic levels in V100 compared to V45 from 1h to 24h (**Figure 6E**). These results indicate that V100 has a higher capability to maintain the ROS at an acceptable level somehow by activating the SOD enzymes activities in contrast to V45. Furthermore, considering the antioxidant role of SOD activity in cold stress tolerance, we genome-wide identified the GmSOD genes in soybean (**Table S3.1**) and predicted their 3D protein structure (**Figure 6A**). To confirm the genetic regulations of SOD, we downloaded the GmSODs expression levels from the RNA-seq database, and found variable expression patterns under cold stress treatment, as shown in the heatmap (**Figure 6C**). Then, we analyzed the gene ontology (GO) terms of these GmSODs. As expected GmSODs were enriched in various oxidative stress-responsive cellular organs, such as mitochondria (21%), extracellular regions (43%), and vacuole (4%). (**Figure 6F**). Subsequently, we analyzed the expression pattern of GmSOD in V100 and V45 under control and 1h and 24h cold stress through qRT-PCR. As expected, the expression levels of selected GmSOD genes such as *GmSOD01*, *GmSOD08*, and *GmSOD1*2 were observed higher in V100 compared to V45 (**Figure 6D**). However, as shown in the heatmap, few GmSODs expression levels were downregulated in cold compared to control in soybean (**Figure 6C**). This situation drew us to the strict posttranscriptional regulation of GmSOD genes by miRNA in soybean. Therefore, we predicted the genome-wide abundance of miRNA families that target the SOD genes in soybean. It was interesting to report that GmSOD genes were regulated by multiple miRNAs or their family members (**Figure 6B**; **Table S3**.2). The most famous abiotic stress-responsive miRNA families that regulated GmSODs were included miR159, miR156, miR167, miR319, miR395, miR396, and miR398 (**Figure 6B**). However, these miRNAs somehow relaxed the GmSODs expression under cold stress conditions to combat produced ROS to protect cellular membrane or organ damage. All these results suggested that SOD antioxidant activity played a protective role and expression of these GmSODs is strictly controlled by their posttranscriptional regulators miRNA to enhance cold tolerance.

### The role of antioxidant POD, related genes and their post-transcriptional regulators in V100 and V45

**Figure 7:**
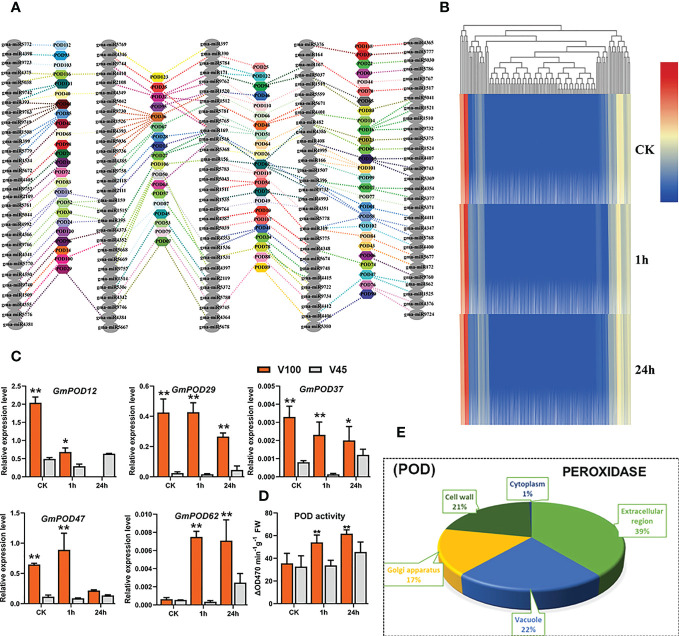
GmPOD genes and posttranscriptional regulation in V100 and V45. **(A)** Post-transcritpional regulation of GmPOD by gma-miRNA. **(B)** RNA-seq expression analysis of GmPOD genes under control **(CK)** and cold stress conditions. Expression data was normalized by log2FC. **(C)** qRT-PCR verification of GmPOD genes in V100 and V45. **(D)** POD enzyme activity in V100 and V45. **(E)** Gene ontology analysis of GmPODs. Data was analyzed with the Statistix 8.1. The ** and * asterisks indicate the significant differences at p-value <0.01 and p-value <0.05, respectively.

Excessive ROS production, such as H_2_O_2_ due to disequilibrium of oxidative reactions under stress conditions, eventually leads to oxidative damage in plants (Zhang et al., 2022). Peroxidases (PODs) are plant-specific glycoproteins are responsible for scavenging stress-induced H_2_O_2_ and transferring downstream stress signaling. Class III peroxidases (PODs) are mainly located in the cell wall and storage vacuoles of plants (Passardi et al., 2005). The POD genes are well known for their physiological and developmental role including cell elongation, cell wall, lignin and suberin formation, ROS scavenging, and protection against abiotic stresses including cold stress (Gabaldón et al., 2005; Wu et al., 2019). Considering the significant role of POD in cold tolerance, we measured the POD antioxidant activity in V100 and V45 in the cold stressed samples for 1h and 24h and control conditions. Cold stress Increased the POD antioxidant activity in both varieties which showed a physiological stress response. Interestingly, compared with V45, the POD enzyme activities were noticed higher in V100 which indicates the robust and decisive response of cold tolerant variety to generated ROS during 1h to 24h stress (**Figure 7D**). As POD genes are activated in response to abiotic stresses and play an antioxidant role in response to cold stress, therefore, we genome-wide discerned the GmPOD genes in soybean (**Table S4.1**). To confirm their role and biological function we performed a GO enrichment analysis of GmPODs. As per expectations, GmPODs were enriched in extracellular regions (39%), vacuole (22%), Golgi apparatus (17%), cell wall (21%) and cytoplasm (1%) in soybean (**Figure 7E**). To learn about genetic regulations of POD activities under cold stressed soybean, we retrieved the GmPODs RNA-seq expression data under cold stress treatment and shown in a heatmap (**Figure 7B**). Heatmap results exhibited that majority of GmPOD genes remained silent. However, with few exceptions, some GmPODs showed a dispensable role by exhibiting the conserved similar relative expression under control and cold stress conditions. Then, we selected a few GmPOD genes and evaluated their relative expression in V100 and V45 under control, 1h and 24h cold stress through qRT-PCR. As per expectations, the relative expression levels of GmPOD genes such as *GmPOD29*, *GmPOD37* and *GmPOD72* were recorded significant higher in V100 compared to V45 which solely indicates the response of the genetic difference in varieties (**Figure 7C**). Interestingly, a large number of GmPODs gene expression remained unchanged under cold stress; possibly, they respond to different stresses or activate at different growth stages in soybean (**Figure 7B**). To find out whether GmPODs are regulated at the post-transcriptional level, for this, we predicted the genome-wide abundance of miRNAs that target and regulate the POD gene expression in soybean. Interestingly, GmPOD genes were regulated by hundreds of miRNAs or their family members (**Figure 7A**; **Table S4.2**). Among these miRNA, abiotic stress-responsive miRNA families that regulate GmPODs including miR156, miR164, miR167, miR171, miR390, miR393, miR408, and miR319 (**Figure 7A**). However, higher expression of some GmPODs under cold stress indicates these genes are regulated by situation mode by miRNAs to ameliorate plant stress effects. These findings suggested that POD have an antioxidant role in protecting cellular damage from stress and their expression is coordinately regulated by a plethora of miRNAs to enhance cold tolerance.

### The role of trehalose (TPS) related genes and their post-transcriptional regulators in V100 and V45

**Figure 8:**
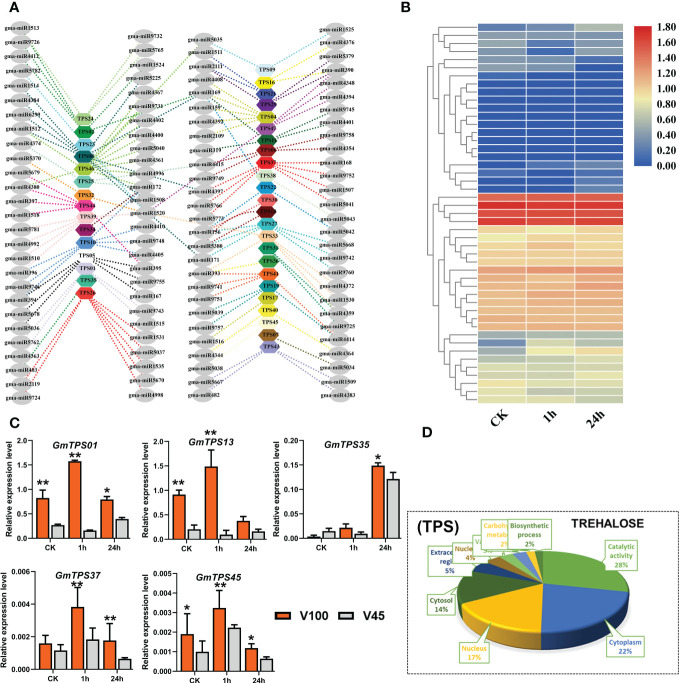
GmTPS genes and posttranscriptional regulation V100 and V45. **(A)** Posttranscritpional regulation of GmTPS genes by gma-miRNA. **(B)** RNA-seq expression analysis of GmTPS genes under **(CK)** and cold stress conditions. Expression data was normalized by log_2_FC. **(C)** qRT-PCR verification of GmTPS genes in V100 and V45. **(D)** Gene ontology analysis of GmTPS. Data was analyzed with the Statistix 8.1. The ** and * asterisks indicate the significant differences at p-value <0.01 and p-value <0.05, respectively.

Cold stress triggers the overproduction of ROS which causes redox imbalance and subsequently accumulation of osmo-protectants in plants (Ding et al., 2019; Mehmood et al., 2021). Trehalose is a non-reducing disaccharide sugar that acts as osmo-protectant molecules induced by numerous abiotic stresses, including cold stress. Interestingly, Trehalose has a dual role not only in supporting plant metabolisms but also acts as a signaling molecule (Kosar et al., 2019; Joshi et al., 2020; Raza et al., 2022). Different studies reported the importance of trehalose in cold stress in different crops; therefore, we selected the GmTPS genes to evaluate their expression response under cold stress in soybean. We performed genome-wide identification and downloaded the trehalose genes and retrieved their RNA-seq expression level under cold stress conditions in soybean (**Figure 8B**, **Table S5.1**). As illustrated in the heatmap, the expression of different GmTPS genes were upregulated in response to cold stress (**Figure 8B**). Then we did qRT-PCR to evaluate the expression differences in V100 and V45 for selected trehalose genes. Interestingly, under cold stress conditions, GmTPS genes were induced as expected, and the expression was significantly up-regulated in V100 compared to V45 which indicated the genetic differential response under cold stress conditions (**Figure 8C**). GO analysis for GmTPS genes showed enrichment of various biological functional terms; most important among them are catalytic activity (28%), cytoplasm (22%), and nucleus (17%) (**Figure 8D**). To further explore the posttranscriptional regulation of GmTPS genes, we genome-wide extracted the miRNA and their family members in soybean. We found that hundreds of miRNAs regulate the expression of GmTPS genes in soybean; among them, well-known miRNAs are also involved including, miR171, miR393, miR397, miR169, miR2111 and miR396 (**Figure 8A**, **Table S5.2**). However, higher expression of some trehalose genes (*GmTPS01*, *GmTPS13* and *GmTPS37*) under cold stress indicates these genes are critical to reducing the cold stress effects in soybean.

### Induced H_2_O_2_ and MDA levels in cold stressed soybean seedlings

**Figure 9:**
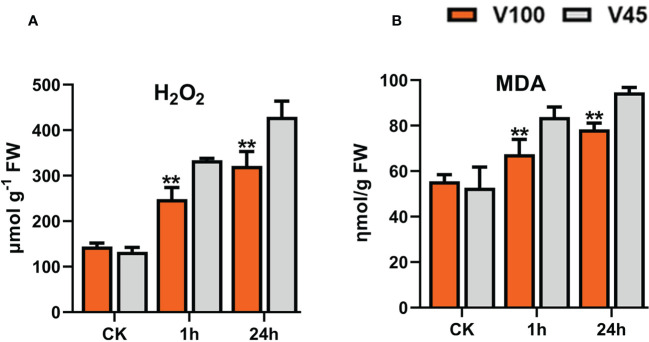
Comparison of H_2_O_2_ and MDA accumulation in V100 and V45. **(A)** H_2_O_2_ levels under control **(CK)** and cold stress in V100 and V45. **(B)**. MDA levels under control **(CK)** and cold stress in V100 and V45. Data was analyzed with the Statistix 8.1. The ** asterisks indicate the significant differences at p-value <0.01.

Cold stress induced the production of different ROS including H_2_O_2_. H_2_O_2_ plays a dual function, such as activating the stress-responsive mechanism; however, excessive H_2_O_2_ produces deleterious impacts on cellular organs and cell membranes. We observed that cold stress increased the accumulation of H_2_O_2_ in V45 in contrast to V100 and control conditions (**Figure 9A**). Similarly, the MDA levels indicate oxidative damage to cells by lipid peroxidation under stress. In our study, MDA content surged in V45 compared to V100, under cold stress respectively (**Figure 9B**). Briefly, cold stress induced the production of H_2_O_2_ and MDA; however, their production was lowered in tolerant variety compared to sensitive variety.

### Effect of cold stress on the expression levels of cold-related marker genes in V100 and V45

**Figure 10:**
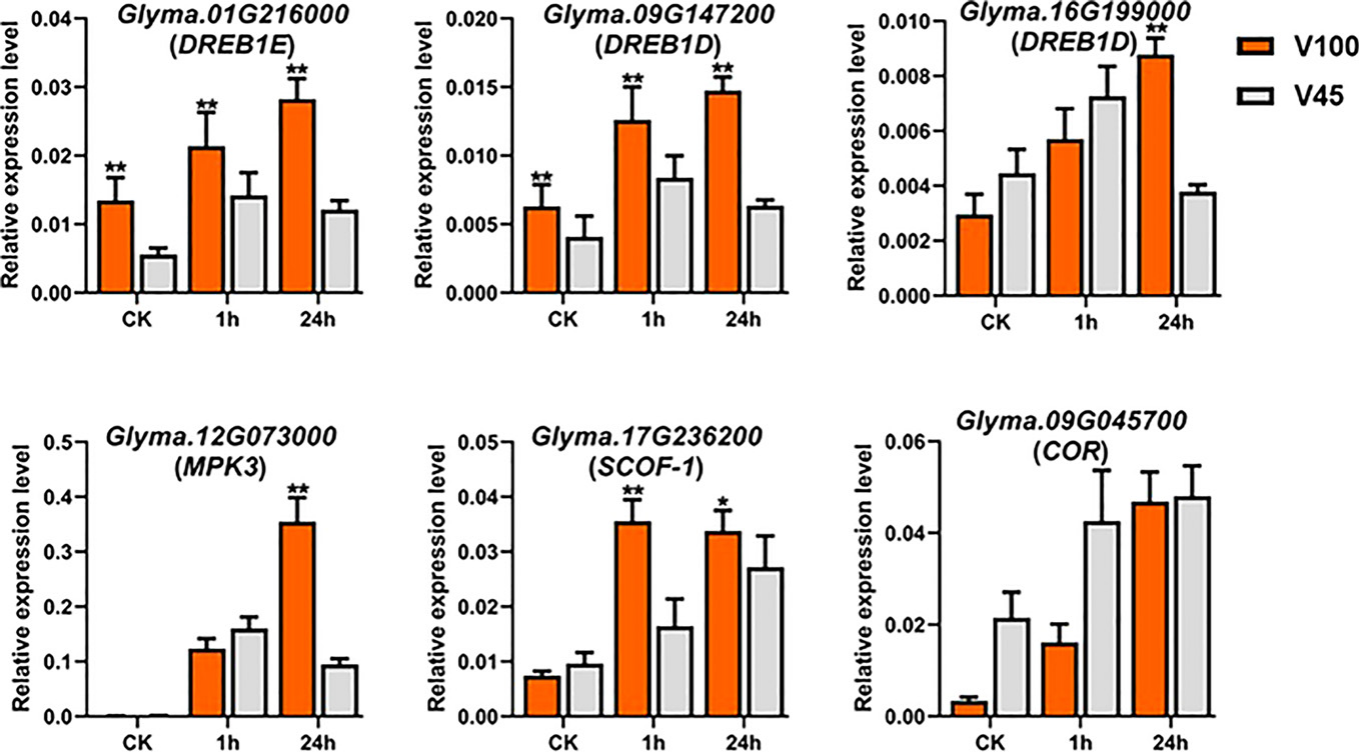
qRT-PCR expression analysis of cold related marker genes in V100 and V45. The time course qRT-PCR was performed to evaluate the expression differences of cold marker genes in cold tolerant V100 and cold sensitive V45. Data was analyzed with the Statistix 8.1. The ** and * asterisks indicate the significant differences at p-value <0.01 and p-value <0.05, respectively.

Soybean genome is enriched with various kinds of cold stress-related CBF/DREBs and COR genes that participate in cold signaling and plant stress response mechanism. Cold stress genes usually work in a complex network and their promoter regions are enriched with stress-responsive binding element sites to regulate the expression of downstream cold genes. To get comprehension insights into the cold-mediated regulation and gene expressions, we attempted the qRT-PCR analysis to observe the expression levels of CBF/DREBs and COR genes in soybean (**Figure 10**). Our qRT-PCR results showed the induction of CBF/DREBs and COR genes in cold-stressed seedlings compared to control conditions (**Figures 10**). Likewise, the expressions of DREB1E, DREB1Ds, SCOF-1 and MPK3 were induced/reduced in cold-stressed seedlings of V100 at different time points compared to V45. Whereas at control conditions, the expression levels of COR genes remained quite similar with few exceptions in both varieties. (**Figures 10**). However, there was considerable expression change in the cold regulated protein (COR) genes under cold stress in V45. Altogether, all the cold-related genes exhibited differential and higher expression levels in the cold stress seedlings of V100 and V45. However, higher induction of COR/DREBS genes along with other improved physiological traits make the V100 more cold tolerant variety.

## Discussion

### Evaluation of soybean germplasm for physiological traits under control and cold stress

Soybean is a multipurpose crop, most vitally as oilseed and protein, grown worldwide and affected by cold stress. In this study, we performed cold stress screening of a large population of vegetable soybean varieties grown in wide geographic latitudes and altitudes, intending to identify cold stress-tolerant varieties for the soybean breeding program. With the advancement of technology and improvement in the biostatistical algorithm, non-destructive methods for (cold) tolerant genotype screening are efficiently applied with high confidence (Wang et al., 2015; Wang et al., 2016a; Yang et al., 2021). Cold stress reduces photosynthesis and affects growth performance. Therefore, we used the non-destructive method of germplasm screening by selecting the photosynthesis-related traits such as qL, Fv/Fm, NPQt, SPAD and phenological trait leaf injury index, leaf thickness under cold stress. Analysis of variance depicted that these traits had high heritability which indicates that these traits are genetically controlled and any difference in these varieties is purely due to their genetics and there is little influence of environment under controlled experiment (**Figure 1B**). The selected photosynthesis traits qL, Fv/Fm, NPQt, SPAD and leaf injury index (Zeng et al., 2021) in our study have been efficiently exploited for the screening and evaluation of drought, salt (Cho et al., 2021; El-Hendawy et al., 2022), and cold (Wang et al., 2016a; He et al., 2019), stress-tolerant crop germplasm and for genome-wide association studies which help identification of important genomic loci/QTLs, and genes. To better correlate these traits, we performed correlation analysis which indicates the higher and significant negative correlation among qL, Fv/Fm and leaf injury index traits which indicates that, indeed, cold stress halted the photosynthesis and caused a cellular injury which was displayed at recovery conditions in the form of leaf injury. Accumulatively, this helps us to identify the cold tolerant (V100) and cold sensitive (V45) soybean varieties.

### Physiological, biochemical and genetic differences between V100 and V45 under cold stress

To better understand the physiological, biochemical and genetic differences between V100 and V45 for cold tolerance capability, we individually evaluated these two varieties. We observed that V100 outperformed in all studied traits such as qL, Fv/Fm, NPQt and leaf injury index compared with V45 for physiological traits as described (**Figures 4A** **–H**). The V100 protects its photosynthesis apparatus by maintaining the significantly higher qL, Fv/Fm and by reducing the NPQt level which indeed helps to maintain a normalized leaf cellular environment and provide protection against leaf injury. Similarly, cold or freezing tolerant poplar maintained higher qL and Fv/Fm by protecting the photosystem (He et al., 2019). In previous studies, comparative RNA-seq analysis showed up-regulation of several photosynthetic genes in response to cold in tobacco and *M. × giganteus* (Spence et al., 2014; Gao et al., 2022). The chloroplast is the primary biosynthesis place of defense-related hormones (Bwalya et al., 2022); therefore, we determined whether photosynthesis genes are involved in cold defense in soybean. The significantly higher expression of photosynthesis-related genes in V100 compared to V45 also indicates these traits are genetically controlled and well preserved in tolerant varieties (**Figure 5C**). The cold stress response is a very complex process, and the response mechanism involves various kinds of ROS such as H_2_O_2_ and membrane peroxidation by MDA. The induction of H_2_O_2_ and MDA is negatively correlated with cold tolerance in plants (He et al., 2021; Raza et al., 2022). Thus, we quantified the H_2_O_2_ and MDA in the cold-stressed soybean; strikingly the contents of H_2_O_2_ and MDA were significantly increased under cold stress. However, significantly lower accumulation of H_2_O_2_ and MDA levels in V100 under cold stress indicates that strong scavenging and ROS control mechanism was activated in cold tolerant variety compared to cold sensitive variety. These results are in accordance with the physiological traits we studied; hence, V100 had higher qL and Fv/Fm levels and had better protection for leaf injury from ROS (**Figure 5H**). The differences in ROS levels in V100 and V45 attracted our attention to the role of activating a strong antioxidant system in response to cold stress. Antioxidative enzymes SOD and POD displayed significant function in the redox stability by ROS scavenging and are generally considered markers of cold tolerance (Mittler, 2002; Liu et al., 2021). Indeed, cold stress enhanced the accumulation of SOD in both varieties however SOD levels were higher in V100 after 24h cold treatment. H_2_O_2_ could act as a signaling molecule to provoke SOD enzyme activity. Under cold stress, the induced SOD antioxidant activity accelerated the conversion of O^2−^ to H_2_O_2_, then efficiently converted into H_2_O and O_2_ by APX enzymes (Liu et al., 2021). Similarly, our POD quantification analysis depicted the enhanced levels in V100; these results are anti-correlated with H_2_O_2_ level. These results indicate the strong induction of the antioxidant system to ameliorate the cold effects in soybean. Therefore, upon cold stress treatment, the membrane lipid peroxidation damage was lower in V100 by strong activation of SOD and POD enzymes which improves the physiological traits same as previously reported in drought stressed sweet basil (Zulfiqar et al., 2021). To understand the genetic mechanism of SOD and POD regulation in cold response in V100 and V45, we genome-wide identified the gene families of GmSOD and GmPOD. GO analysis of these genes’ families showed the enrichment in various stress-responsive biological terms. Most importantly SOD genes family is enriched in the following top GO terms such as extracellular region (GO:0005576), mitochondrion (GO:0005739), cytosol (GO:0005829), vacuole (GO:0005773), thylakoid (GO:0009579) and nucleus (GO:0005634) (**Figure 6F**). These results showed that SOD genes are activated in multiple organs to remove excessive ROS within the cell boundary to maintain and protect the cell structure from cold damage. In contrast to SOD genes, the POD genes family enriched in the following top GO terms response to stress (GO:0006950), cell wall (GO:0005618), Golgi apparatus (GO:0005794), cytoplasm (GO:0005737), vacuole (GO:0005773), and extracellular region (GO:0005576) in soybean. The differential enrichment of these terms in SOD and POD genes further suggested that different genes and their final products coordinated to reduce the detrimental effects of cold stress conditions in soybean. We compared the expression levels of GmSOD and GmPOD genes through qRT-PCR among V100 and V45, and between previously reported RNA-seq data under cold stressed soybean for 1h and 24h. Interestingly, only a few SOD and POD genes were upregulated in response to cold treatment in soybean. The higher expression of selected GmSOD and GmPOD genes in V100 compared to the previous study indicates genetic differences in the response of varieties under cold stress. Similarly, SOD genes respond to multiple stresses including, salt, drought, and cold in different plants (Song et al., 2018; Su et al., 2021). In previous studies, overexpression of POD genes enhanced the tolerance to cold (Llorente et al., 2002), salt (Jin et al., 2019), drought (Kumar et al., 2012; Aleem et al., 2022), bacterial pathogen (Wally and Punja, 2010), and fungal disease (Mika et al., 2010). These studies indicate that SOD and POD gene families are inbuilt with diverse functions and their family members respond selectively and cooperatively in plants. To further analyze the cooperative role between antioxidants, metabolites and osmo-protectants during cold stress in soybean, we retrieved and analyzed the GmTPS gene family. Trehalose act as a molecular cushion and transfer downstream signals to activate the stress responses. Similarly, exogenous application of Trehalose improved stress resilience such as cold (Fu et al., 2020; Raza et al., 2022), salt (Yang et al., 2014), and drought (Zulfiqar et al., 2021). Due to its smaller molecule size trehalose is easily absorbed through cell pores and enhanced the stress response to reduce oxidative damage by activating the proline, SOD, POD, CAT, and APX enzymes and stress-responsive gene expression (Liu et al., 2021). Gene ontology analysis revealed that trehalose genes are enriched in following terms; catalytic activity (GO:0003824), carbohydrate metabolism (GO:0005975), cytoplasm (GO:0005737), nucleus (GO:0005634), extracellular region (GO:0005576), and vacuole (GO:0005773) (**Figure 8D**). The enrichment of trehalose genes in these biological terms indicate the important role in energy conversion and maintenance of internal organ in cell structure from oxidative stress and cold damage. Thus, in this study, we fetched the RNA-seq expression data of trehalose genes and found that most of the genes showed variable and higher expression in cold stressed soybean (**Figure 8C**). Similarly, our qRT-PCR expression analysis identified the enhanced expression of selected GmTPS genes in cold stressed V100 and V45. The expression level of genes *GmTPS01*, *GmTPS13* and *GmTPS37*, was found higher in V100 compared to V45 under cold stress; however, *GmTPS37* and *GmTPS37* had no significant differences in V100 compared to V45 at normal conditions. Thus, trehalose genes increased cold tolerance by ameliorating oxidative stresses and activating the antioxidant system in cold-tolerant soybean.

### Role of posttranscriptional regulators and cold marker genes under cold stress in soybean

The miRNAs proved important posttranscriptional regulators of target genes expression and a plethora of genome-wide studies had identified miRNA in soybean that responds to multiple environmental stresses (Sun et al., 2015; Ramesh et al., 2019). In the present study, we identified multiple miRNAs families targeting GmSODs, GmPODs and GmTPS genes. Interestingly, many miRNAs regulate the expression of a single gene in soybean. Similarly, in cotton, the GhSODs were regulated by 20 miRNAs (Wang et al., 2017). In our study, previously reported miRNA that regulates expression of SODs genes were also procured, which showed the conserved role of these miRNA in different plant species such as Arabidopsis miR398 mediates regulation of AtSOD genes (Beauclair et al., 2010) were also observed in soybean. Furthermore, we predicted hundreds of miRNAs that regulate the expression of GmPOD genes (**Figure 7A**, **Table S4.2**). Few of these miRNAs have been previously functionally validated for their stress response in various plants such as miR397 for cold stress in Arabidopsis (Dong & Pei, 2014). To identify the role of miRNA in trehalose gene regulation, we predicted several well-known miRNAs that played a significant role in plant stress adaptation such as miR159, miR169, miR172, miR390, and miR394. These miRNAs have been validated for a different role in addition to abiotic stresses such as miR159 regulating the fatty acid metabolism in rapeseed (Wang et al., 2016b). The miR172 participated in transition from development to flowering (Zhu and Helliwell, 2011). The miR396 enhanced cold tolerance (Zhang et al., 2016), and miR394 increased, cold, drought and salinity tolerance (Song et al., 2013; Song et al., 2016). It was interesting to note that we also found that multiple miRNA families commonly targeted GmSOD, GmPOD and GmTPS genes. These results also suggested that, by modifying the expression of single miRNA it could be possible to change the expression of genes that belong to different gene families and involved in different stresses in soybean. As previous studies identified that antioxidant, metabolites and COR genes jointly regulate stress response and total fitness of plant is dependent on their functionality. Our qRT-PCR analysis for well-studied cold responsive ICE-CBF/DREBs-COR signaling pathways gene (Ding et al., 2019; Kidokoro et al., 2022) revealed the activation of COR pathways in cold stressed soybean. Our qRT-PCR results revealed that cold stress treatment for 1h and 24h substantially increased the expression levels of DREB1E, DREB1Ds, SCOF-1, and MPK3 genes in cold-stressed V100 compared to V45 and control plants (**Figure 10**). These results provide insights that endogenous activation of trehalose genes involved in the transcriptional regulation of CBF/DREBs and CORs genes which act in a concert with antioxidant system in soybean to dispose of the effects of cold stress. Similar findings also reported in Arabidopsis and rapeseed where different metabolites activate the expression of CBF/DREBs and COR genes under cold stress (Shi et al., 2015; Raza et al., 2022).

**Figure 11:**
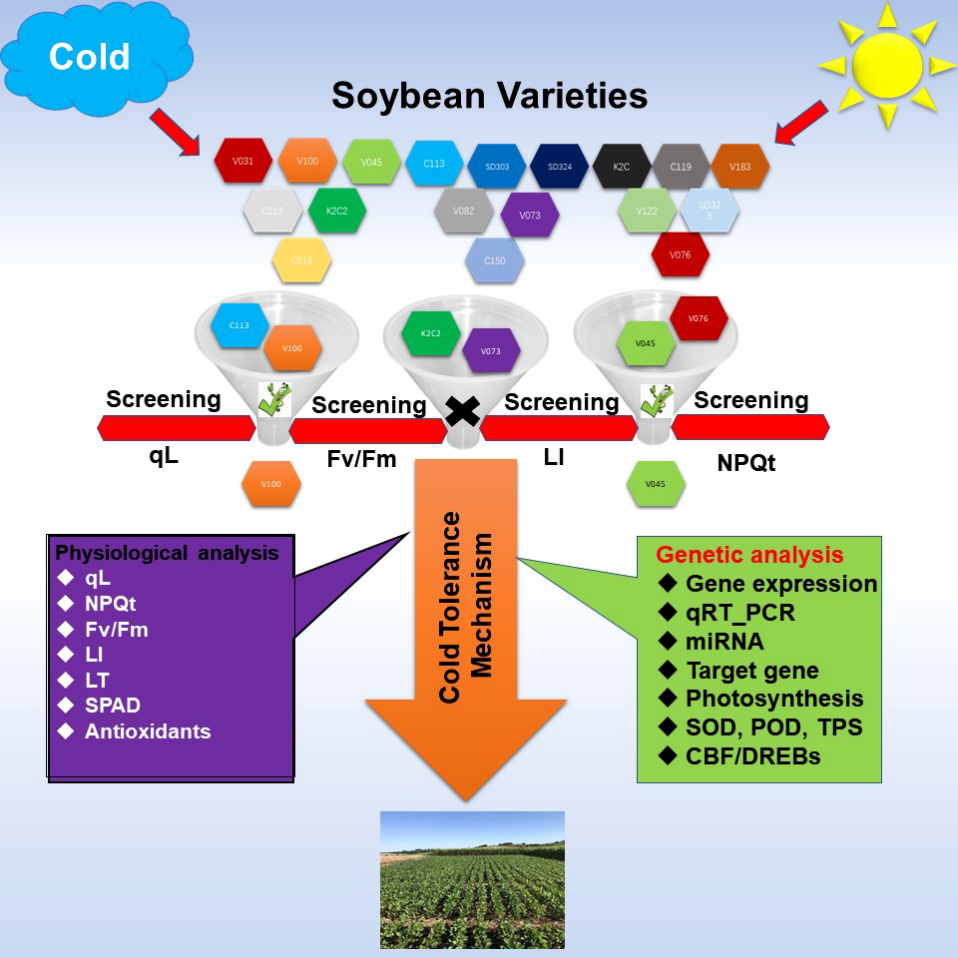
Methodology adopted to identify the cold tolerant germplasm in Soybean. Soybean varieties were screened under control and cold stress conditions to identify the cold tolerant germplasm based on physiological and phenological traits. Physiological traits such as qL, Fv/Fm, NPQT (non‐photochemical quenching), SPAD value, antioxidants enzymes activity such as SOD, POD, and H_2_O_2_ and MDA level were measured. Phenological leaf injury (LI) and leaf thickness were recorded and analyzed. For genetic analysis, the expression levels of cold stress related genes and cold marker genes were analyzed through RNA-seq and qRT-PCR analysis. The posttranscriptional regulators of these genes were excavated. All these analysis combinedly help to explain the major difference for cold tolerance and cold sensitivity in soybean varieties.

## Conclusion

In the current study, we screened a large panel of soybean varieties under cold stress to identify the cold tolerant and cold sensitive varieties. The strategy adopted here included the non-destructive phenological and physiological approaches such as photosynthesis-related traits and leaf injury index as a screening method (**Figure 11**). Thus, using these methods, we identified two cold tolerant (V100 and V037) and cold sensitive (V45 and V004) varieties. Cold tolerant V100 and cold sensitive V45 were selected for further analysis to identify the differences in their genetic response. These two varieties were evaluated for photosynthesis-related traits, leaf injury index, relative expression of photosynthesis, SOD, POD, and TPS related genes through qRT-PCR. Additionally, we identified the posttranscriptional regulators miRNAs of GmSOD, GmPOD and GmTPS genes. Furthermore, ROS such as H_2_O_2_ and MDA and accumulation of antioxidants SOD and POD levels were quantified. Finally, qRT-PCR for cold marker genes was performed. Altogether, our results exhibited that most of these genes significantly responded to cold stress treatment and differences in V100 and V45 cold tolerance lies in their genetics and response mechanism involving activation of various kinds of antioxidant genes, their byproducts, metabolites and less accumulation of ROS and MDA to protect the photosynthesis apparatus and cellular injury. The cold stress response is a complex mechanism; it involves various kinds of stress-responsive pathways and better coordination between these pathways helps to ameliorate the cold stress effects. Thus, additional functional genomic work is direly needed to confirm the role of these pathways in soybean cold tolerance. We identified cold tolerant and sensitive soybean varieties which could be used for comparative transcriptomic, metabolomic, and proteomics studies and as a crucial genetic resource for future environment resilient breeding programs.

## Data availability statement

The original contributions presented in the study are included in the article/**Supplementary Material**. Further inquiries can be directed to the corresponding author.

## Author contributions

MH, and HL conceived the idea and wrote the manuscript. MH, LS, and HG performed the experiments. CF, LS, PS, XS, YJ and KX helped in the literature searches and data analysis. WZ, YZ and HL supervised the work, reviewed, edited the manuscript, and manage funding resources. All authors contributed to the article and approved the submitted version.

## Supplementary material

Supplementary Table 1 Soybean varieties evaluated for cold tolerance.

Supplementary Table 2 qRT-PCR primers used in this study.

Supplementary Table 3 GmSOD genes RNA-seq expression and their target miRNAs.

Supplementary Table 4 GmPOD genes RNA-seq expression and their target miRNAs.

Supplementary Table 5 GmTPS genes RNA-seq expression and their target miRNAs.
